# I like therefore I can, and I can therefore I like: the role of self-efficacy and affect in active inference of allostasis

**DOI:** 10.3389/fncir.2024.1283372

**Published:** 2024-01-22

**Authors:** Valery Krupnik

**Affiliations:** Department of Mental Health, Naval Hospital Camp Pendleton, Camp Pendleton, Oceanside, CA, United States

**Keywords:** active inference, allostasis, self-efficacy, affect, interoception, fatigue, depression, affective charge

## Abstract

Active inference (AIF) is a theory of the behavior of information-processing open dynamic systems. It describes them as generative models (GM) generating inferences on the causes of sensory input they receive from their environment. Based on these inferences, GMs generate predictions about sensory input. The discrepancy between a prediction and the actual input results in prediction error. GMs then execute action policies predicted to minimize the prediction error. The free-energy principle provides a rationale for AIF by stipulating that information-processing open systems must constantly minimize their free energy (through suppressing the cumulative prediction error) to avoid decay. The theory of homeostasis and allostasis has a similar logic. Homeostatic set points are expectations of living organisms. Discrepancies between set points and actual states generate stress. For optimal functioning, organisms avoid stress by preserving homeostasis. Theories of AIF and homeostasis have recently converged, with AIF providing a formal account for homeo- and allostasis. In this paper, we present bacterial chemotaxis as molecular AIF, where mutual constraints by extero- and interoception play an essential role in controlling bacterial behavior supporting homeostasis. Extending this insight to the brain, we propose a conceptual model of the brain homeostatic GM, in which we suggest partition of the brain GM into *cognitive and physiological homeostatic* GMs. We outline their mutual regulation as well as their integration based on the free-energy principle. From this analysis, affect and self-efficacy emerge as the main regulators of the cognitive homeostatic GM. We suggest fatigue and depression as target neurocognitive phenomena for studying the neural mechanisms of such regulation.

Living organisms, including humans, evolved because of and for survival and procreation. These goal states, however, cannot be hard-wired in the brain because they are dynamic, context-specific, and behavior-dependent. Instead, organisms use proximal internal states indexed by homeostasis and allostasis for guidance (McEwen, 2000; Sterling, 2004; Peters et al., 2017). An in-depth analysis of the relationship between the two concepts can be found elsewhere (Corcoran et al., 2020). Here, we refer to homeostasis as a process, by which an organism maintains its parameters at its set points. Allostasis describes a process, by which an organism adjusts its parameters to adapt to anticipated change in demands (often environmental) placed on it (McEwen, 2000; Sterling, 2004). A common example is rising blood pressure before we stand up. If allostasis results in an adjusted stable state, it becomes a new homeostatic state. Therefore, we also use homeostasis as an umbrella term for homeo- and allostasis similarly to the view of allostasis as “a process that supports homeostasis” (McEwen and Wingfield, 2003, p 3). Homeostatic set points are hard-wired and optimized through evolution for optimal performance in the game of life: using environmental resources to survive and procreate, but adaptive behaviors are required to maintain them through developmental and environmental change.

In brain-endowed organisms, the brain controls the organism’s behavior but has no direct access to its homeostatic states, such that bodily states of, e.g., hunger or cold are sensed by peripheral organs and neurons that pass that information onto the brain [although, certain vitally important for brain functioning parameters such as the levels of oxygen and glucose are directly sensed by the brain neurons (Song et al., 2001; Neubauer and Sunderram, 2004)]. For the most part, the brain relies on the readout by the interoceptive brain network which signals how close or far the organism is from its set points, or in other words, how low or high its stress/dyshomeostasis^1^ is. Stress dynamics are associated with emotional reactions, negative emotions reflecting growing and positive—decreasing stress (Solomon, 1980). Accordingly, to maintain homeostasis organisms try to occupy “feel good” (low stress) states. Why then do we often act in ways that make us feel bad or do not always act in ways that make us feel good? The challenge here is that to *stay* in a low stress state an organism has to maintain its optimal functioning while meeting the environmental demands, not an easy task given the complexity and dynamic nature of the environment of life.

To be in a “feel good” state an organism has to accomplish several cognitive tasks. It needs to know what makes it feel good, that is to have a model of the sentient/physiological self. It needs to know where in its environment it may feel good, for which it needs to have a model of the environment and its affordances. It needs to know what behaviors (action policies) to execute to place itself in a desired state, so it needs a model of the behavioral self. It also needs to estimate how likely the selected action policy is to result in the expected outcome, which means it has to have a meta-cognitive model of the cognitive self. These cognitive operations have to be optimally integrated to result in a coherent successful behavioral strategy. Active inference theory (AIF) provides an account for such integration (Pezzulo et al., 2015).

In this paper, after a brief primer on AIF, we consider the simplest known behavior, bacterial taxis, to elucidate the evolutionary blueprint for embodiment of AIF in molecular mechanisms. We then extrapolate this blueprint onto the hierarchical generative model (GM) of the brain and propose a conceptual model of the functional integration of its different levels and sub-models. Specifically, we suggest partitioning the brain GM into cognitive and physiological homeostatic GMs with affect and self-efficacy (SE) as the mechanisms of their integration and redundancy as its principle. We argue that such partition provides for more robust and flexible regulation of animal behavior as organisms adapt to often competing demands on their resources. We then suggest fatigue and depression as, respectively, experimental and clinical models useful for the identification and study of neural networks involved in the integration of the two homeostatic GMs. Definitions of main terms used in the paper are appended in the glossary.

## Active inference

Active inference is part of the predictive processing paradigm which regards information-processing systems including living organisms as predictive machines. In AIF, organisms embody generative models of their external and internal environments that make inferences (also called predictions, or hypotheses) about the causes of sensory sensations and act on the environments to confirm the inferences by receiving sensations that the model predicted (Friston, 2010; Friston et al., 2017). This represents circular causality, e.g., I expect to see a period at the end of this sentence and move my eyes accordingly to confirm it. Such GMs are thought to have a hierarchical structure (Badcock et al., 2019a), where higher-level units signal their predictions down the hierarchy to sensory organs/receptors that receive signals from the environment and send them in a counterflow up the hierarchy. Predictions about sensory input are based on previously learned or innate (e.g., genetically determined) beliefs about regularities in the environment and are called *priors*. The variance between a prediction and actual input is called *prediction error*. The model constantly updates itself by minimizing prediction errors. Since priors, predictions, and prediction errors can be viewed as probability density functions (in the brain, encoded by neuronal firing), the model is believed to update itself according to Bayesian statistical rules. The better a model minimizes its prediction errors the more accurate it becomes, thus increasing its chances to occupy states generating predicted and sought sensations, which is the AIF meaning of adaptation (Badcock et al., 2019b).

There are two ways for a GM to minimize its prediction errors: (a) through perceptual inference by adjusting its priors and, consequently, predictions to bring them closer to the environmental input, or (b) through active inference by adjusting the organism’s behavior and/or properties to manipulate the incoming sensory stimuli to bring them (statistically) closer to the predictions. The two strategies are usually integrated into an evolving perception-action cycle. To explain the reason behind the need to minimize prediction errors and to link it to first principles, the free-energy principle has been proposed (Friston, 2010). It stipulates that for an information-processing system to preserve its integrity and avoid decay it must avoid sensory surprising states or high informational entropy which is a long-term average of surprise. Variational free energy is an information-theoretic quantity setting the upper bound on the system’s total surprise. Therefore, systems avoid decay by reducing their free energy, which is achieved by minimizing the cumulative prediction error. The free-energy principle has been proposed as a “unified brain theory” (Friston, 2010) since it presumes that all brain functions can be traced to the imperative of reducing its free energy. In addition, by minimizing its cumulative prediction error, the brain maximizes its GM’s evidence, which makes self-evidencing a corollary to the free-energy principle (Hohwy, 2016).

To act on its environment in a way that minimizes its free energy, a GM needs to select an action policy that reduces the *expected* free energy since its quantity can only be confirmed *after* the action. The concept of expected free energy leads to the notion of temporally deep GMs that, based on past learning, plan and execute action policies meant to reduce the free energy in the future (Friston et al., 2018). In temporally deep GMs, the predicted endpoint sensory state can be separated from the present by a lengthy chain of cognitive and behavioral acts.

*Precision-weighting* is a concept central to AIF since GMs are commonly formalized in Bayesian statistics, where priors, predictions, sensory input, and prediction errors are viewed as probability functions whose inverse variance is called precision (Clark, 2015). In this view, a model with a highly precise prior will weight a disconfirming sensory input highly, leading to a high-precision prediction error. The precision of GM’s priors and predictions is the measure of the model’s confidence including confidence in its policies (Friston et al., 2013).

## Bacterial taxis as an example of molecular active inference

Bacterial chemotaxis represents the arguably simplest known behavior whose mechanisms are well understood and molecular players are known (as reviewed, e.g., in Stock et al., 2002; Wadhams and Armitage, 2004). It is also the longest-evolved behavior that has supported adaptation of the most evolutionary successful life domain. This makes it valuable for examining the evolutionary design and logic of active inference embedded in the bacterial cell. Bacterial chemotaxis has been framed in AIF terms before, and its computer simulation was developed (Corcoran et al., 2020), although without taking into account its molecular mechanisms which are fairly complex. Because of this complexity, the molecular machinery of chemotaxis was dubbed “probrain” (Stock et al., 2002).

**Figure 1:**
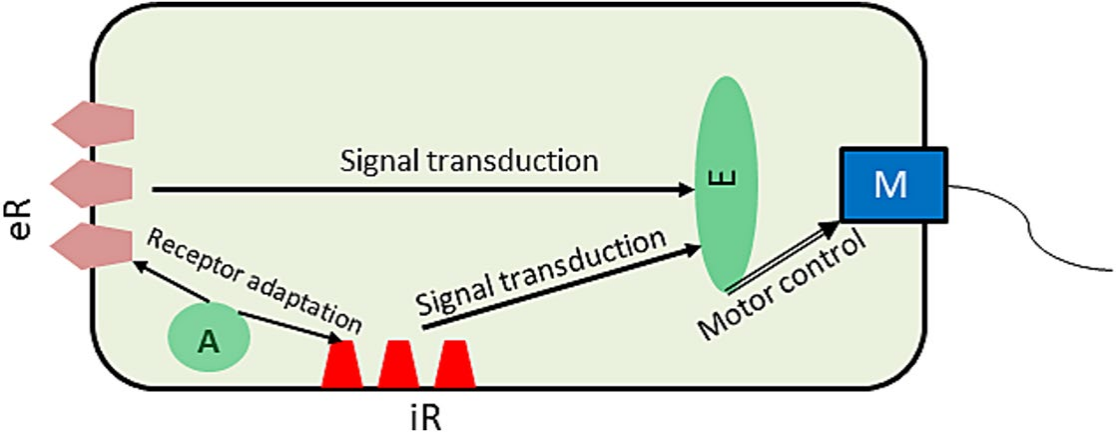
A simplified schematic of molecular mechanisms of bacterial chemotaxis. Information from receptor clusters is transduced to the effector protein which summates it to control the flagellar motor switch and thus the bacterial motion. The adaptation protein regulates excitability of the receptors by methylating them in an activity-dependent manner, so that they respond only to change in their ligand’s levels. eR, exteroceptive receptor cluster; iR, interoceptive receptor cluster; A, adaptation protein; E, effector protein; M, flagellar motor. The arrows show the direction of signaling.

Considering a bacterium as a GM, its core priors determine the homeostatic range of its energy and the chemical content of its body conducive to effective metabolism through the bacterium’s developmental phases. These priors constitute the bacterium’s model of itself or *self-model* which makes dynamic predictions according to the bacterium’s life-cycle stages: growth, DNA replication, division, or sporulation. In the bacterial GM, priors are preset by evolution and encoded in the genome, whereas predictions depend on the bacterium’s stage of the life cycle and environmental conditions. When the priors are at variance with the body parameters, the bacterium finds itself in a surprising sensory state with increased free energy. Bacteria receive sensory input through bacterial membrane receptors, both intero- and exteroceptive (Figure 1).

Cellular energy is monitored by interoceptive receptors sensing change in the redox state of the components of the membrane metabolic cascade called electron transport system as well as change in the energy properties of the membrane itself such as its proton motive force (Alexandre et al., 2004). Decreased energy generates a prediction error that is then suppressed by acting on either the internal environment by hydrolyzing adenosine triphosphate to release its energy or the external environment by moving to a place richer in nutrients. To accomplish that, the bacterium needs to have models of its internal and external environments. These models are encoded in its signal transduction pathways, which we discuss in more detail below, as they relate to bacterial chemo and energy taxis (Figure 1).

**Figure 2:**
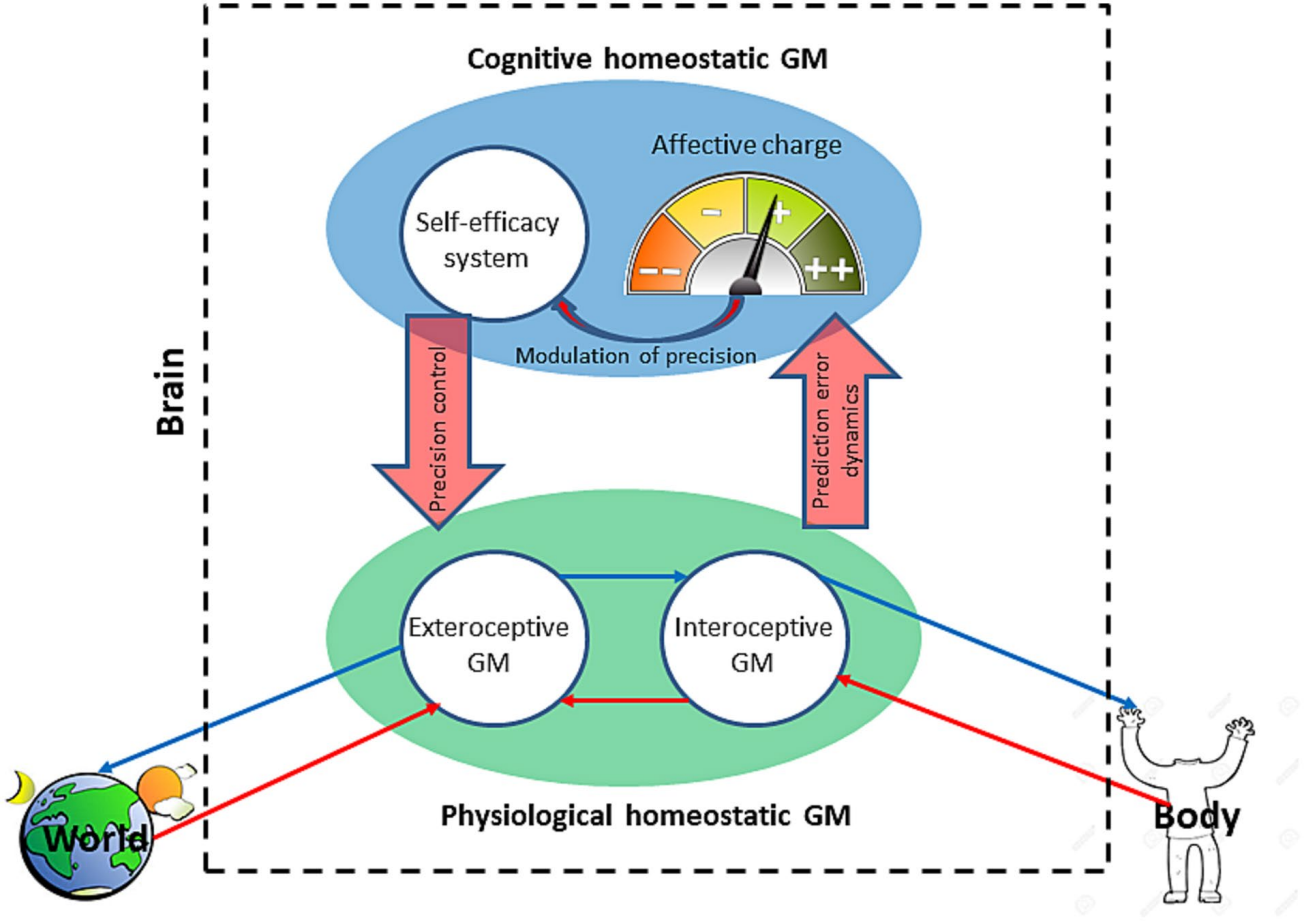
The integrated brain homeostatic generative model. The model is an autoregulatory closed loop that comprises the physiological (green) and cognitive (blue) homeostatic generative models (GM) with the latter hierarchically above the former. The physiological model comprises an exteroceptive GM of the world and interoceptive GM of the body. Prediction error dynamics of the physiological homeostatic GM are signaled to the cognitive homeostatic GM as an affective charge. The affective charge modulates the precision of the self-efficacy system which, in turn, regulates the precision of the physiological homeostatic GM. Thin blue arrows indicate predictions, and the red ones—prediction errors.

Chemotactic receptors bind external nutritious (attractants) and noxious (repellents) molecules, and signal change in their concentration to the effector protein which regulates bacterial movements. Such signals inform the GM of how close the levels of these molecules are to the predicted range. When a bacterium finds itself outside the range (increased prediction error), it seeks and move to a better environment, but how does it know where to go? Flagellar bacteria (such as *E. coli*) have two kinds of motion, tumbling and swimming (Berg, 2004) controlled by their flagellar motor (Figure 2). Sensing an unfavorable condition: no increase in attractants or/and increase in repellents, they tumble randomly sampling the environment in all directions. Once they tumble upon a direction with a favorable change: increased attractants or/and decreased repellents, they swim along the gradient. As a result, their motion proceeds along a “run-sample-run” trajectory. Such a strategy represents the exploration-exploitation cycle of AIF (Kaplan and Friston, 2018). The way a bacterium computes the relative quality of positions in the environment is by adaptation of their receptors. The adaptation protein (Figure 2) receives a signal from the receptor and modifies its sensitivity through methylation, so that the receptor adapts to the current concentration of its ligand and only responds, if the ligand’s concentration changes. The mechanism of adaptation ensures that bacteria always seek out gradients, positive for attractants and negative for repellents by sampling their concentration through tumbling. This allows the bacterial GM to predict their likely gradients and fulfill this prediction by swimming along the gradients until an increase in prediction error (and free energy) calls for tumbling, whereas decreasing prediction error will make for a smoother trajectory with less frequent tumbling.

The precision of molecular GM is encoded in the properties of its signaling molecules. For example, as mentioned above, sensitivity of membrane receptor clusters depends on the level of their methylation, which can be seen as analogous to the regulation of synaptic gain in nervous system. Likewise, the signaling activity of the effector protein can be regulated by its acetylation (Ramakrishnan et al., 1998). If prediction error precision is overweighted, the GM may be highly reactive to environmental noise and may become disoriented in a chemically noisy environment (all tumble and no swim). Should prediction errors be underweighted, the GM may be slow to react to environmental change and may find itself in a noxious environment.

An especially important for this paper feature of the bacterial GM is the integration of its two component models: the model of self and the environment. The former is updated by interoceptive and the latter—exteroceptive receptors. Integration happens at the level of the effector protein which summates signals from all the receptors and transduces the result to the flagellar motor (Figure 2). The result is determined by the relative precision of intero- and exteroceptive signals. Such integration is necessary to optimize bacterial AIF in a complex dynamic environment with sometimes contradictory properties. For example, an aerobic bacterium may find it necessary to avoid a nutritiously rich environment if it is also low on oxygen, without which the cell would be depleted of energy. Thus, the molecular self-model constrains the model of the external environment in the service of minimizing the cumulative prediction error and, consequently, of the GM’s free energy. This design may appear redundant; after all, a favorable nutritious environment is supposed to provide for a high energy internal state. However, such redundancy may result in robust and flexible behavior, where energy- and chemotaxis complement and regulate each other. Despite (or perhaps because) its simplicity, the described molecular AIF allows for an effective behavioral adaptation supporting, as mentioned earlier, the most successful domain of life. Extrapolating this evolutionary logic to brain-endowed organisms, we next explore the integration of the models of self and the environment by the brain GM.

## The brain integrative generative model of stress and homeostasis

Similar to bacterial GM, the brain GM’s primary function is energy regulation through homeostatic mechanisms (Barrett et al., 2016). Unlike the unicellular GM, the brain needs to integrate three component GMs: an exteroceptive model of the world, an interoceptive model of the body/physical self, and a model of cognitive self, since the brain is separated (physically and statistically^2^) from both the world and (for the most part) the body. Their optimal integration means the minimal free energy of the whole brain GM (Figure 2). The integrated model is a cognitive hierarchy with self-conscious domain-general cognitive processes at the top and domain-specific sensory processing at the bottom. The brain GM, as mentioned earlier, is temporally deep, predicting states of the self and environment as consequences of future events and actions, and basing these predictions on prior beliefs. Minimization of the model’s free energy results in the organism’s avoidance of phenotype-incongruent states of sensory surprise. In application to the interoceptive GM,^3^ that means avoidance of stress or dyshomeostasis.

The theory of homeostasis and allostasis has been recast in AIF terms (Peters et al., 2017; Corcoran et al., 2020). According to it, in response to an environmental challenge, the organism’s GM predicts a disturbance of its homeostasis, i.e., dyshomeostasis, and through autonomic reflexes makes an allostatic transition to a new state (Sterling, 2012). Thus, the interoceptive GM is at the same time the organism’s model of stress, guiding its stress response AIF, which has been considered on a continuum from normative to traumatic stress response (Krupnik, 2019, 2020b).

The interoceptive GM encodes priors and predictions for the organism’s metabolic parameters, determining their set points’ probability distribution. For a successful stress response, that is to stay within a state space compatible with life, the organism needs to remain within its allostatic range determined by its allostatic priors (Atzil and Barrett, 2017). Such priors have been conceptualized as allostatic self-efficacy (Stephan et al., 2016). Self-efficacy is a concept from social learning theory that denotes an agent’s confidence in achieving a desired (“predicted,” in AIF terms) outcome (Bandura, 1977). Allostatic SE determines the interoceptive GM’s confidence in how far it can deviate from its homeostatic set points, which is a measure of the organism’s capacity to withstand stress. Stress response happens in a particular environment and thus requires an adaptive behavior to combine external and internal affordances to accomplish stress reduction. This necessitates integration of the interoceptive and exteroceptive GMs into a physiological homeostatic GM (Figure 2). The exteroceptive GM models the organism’s external environment, making inferences about its causal regularities, thus predicting its properties. Its integration with the interoceptive GM creates an organism-in-the-environment model predicting behaviors likely to serve the organism’s homeostatic needs. In a bacterial cell, its interoceptive model of energy set points is integrated (through the effector protein, Figure 1) with its exteroceptive model of attractant and repellent gradients, resulting in an adaptive chemotactic AIF.

An example can be helpful to illustrate the mechanics of allostatic AIF of more complex behaviors. As one is faced with the stress of a physical effort such as catching a bus leaving the station, his GM predicts energy expenditure by the muscles and thus a deviation from homeostasis. This will generate an expected prediction error, relative to the homeostatic priors, and an expected increase in the free energy. The expected prediction error can then be minimized by allostatic predictions and their fulfillment through the autonomic reflexes of increasing heart rate and blood flow to the muscles. This chain of events will result in the allostatic transition necessary to carry the allostatic load of the task. Once the muscles are at work, the allostatic dynamics change again. The muscle and organism’s physiology starts drifting away from the homeostatic set points through, e.g., decrease in oxygenation and glycogen storage, change in the electrolyte levels etc. (Davis and Walsh, 2010). This generates real-time prediction errors, relative to the homeostatic priors, leading to increasing free energy the farther from its homeostatic set points the organism gets. The prediction errors are then minimized by AIF generating the feeling of fatigue and by decreasing the allostatic load by slowing down the physical activity (Greenhouse-Tucknott et al., 2022). Such dynamics may set up a conflict between the higher-level counterfactual GM predicting exertion and the lower-level *sensory near* homeostatic GM predicting rest (see Corcoran et al., 2020 for a philosophical analysis of allostatic vs. homeostatic AIF).

In the example of chasing a bus, the inference is that taking the bus will reduce the expected prediction error of, for example, being late or not reaching the agent’s predicted destination at all. This exteroceptive GM stands to contradict the described above interoceptive GM accumulating the prediction error of allostatic load and predicting fatigue. How does the agent reconcile such a contradiction and integrate the extero- and interoceptive GMs?

One possibility is that the choice of action policy—rest vs. pursuit of the bus—is determined by the balance of the contradictory models’ confidence. In AIF, confidence refers to the precision of an agent’s belief that a certain behavior will achieve the predicted outcome (Friston et al., 2013), which is tautological with SE. Thus, confidence or SE is a function of the precision of an action policy (henceforth, confidence and SE will be used interchangeably to make the narration more intuitive). Relying on the balance of (molecular) precisions is how bacterial AIF, described in the previous section, is optimized. However, this mechanism may not always be optimal for more complex GMs such as the brain. The reason for this is the brain GM’s greater depth such that the farther the model reaches from its sensory experience in both time and context, the more counterfactual and less accurate it becomes. This makes the task of minimizing the expected free energy of the integrated GM more challenging. In the bus example, a hyper-confident exteroceptive model may “push” the organism beyond its allostatic range, causing damage (a heart attack in an extreme case), whereas a hyper-confident homeostatic GM may have the agent underperform. Either strategy would, in the end, fail at optimizing the integrated GM. This means that the lateral (bacteria-like) mechanism of integrating the intero- and exteroceptive GMs into a physiological homeostatic GM via mutual constraints may be suboptimal for complex decision-making.

We propose the concept of cognitive homeostasis (and cognitive homeostatic GM)^4^ as an additional higher order mechanism of optimization of the integrated brain GM (Figure 2). Such regulatory redundancy, as noted above for bacterial chemotaxis, is expected to confer greater robustness and flexibility on the system’s behavior. Cognitive homeostasis controls the integrated GM’s free energy which serves as its dynamic homeostatic variable. In this sense, cognitive homeostasis functions as the hierarchically highest metacognitive level of self-modeling since it models the GM’s overall performance. Framing it as homeostasis provides a conceptual link to physiological homeostasis thus extending the free-energy principle from a first principles-based “unified brain” theory (Friston, 2010) to a unified theory of organism. There is a notable distinction to cognitive homeostasis. Whereas the range of physiological variables is determined by evolutionary and developmentally preset homeostatic priors, the brain GM’s free energy is a quantity that, according to the free-energy principle, is continuously minimized but can never be zero (Friston, 2009).

Partition into cognitive and physiological homeostasis implies likewise partition into cognitive and physiological dyshomeostasis/stress, where cognitive stress means any increase in the brain GM’s free energy, and physiological stress is an increase in the free energy caused by interoceptive prediction error. Whereas physiological homeostatic GM receives sensory information directly through corresponding sensory channels, cognitive homeostatic GM has to be able to predict and track the dynamics of the free energy through metacognitive mechanisms. We suggest that SE and affect play the role of such mechanisms, where SE regulates the dynamics of the precision of action policies, and affect provides “sensory” information about the dynamics of the GM’s cumulative surprise. This way, the system of SE can “sense” and be updated through the system of affect about the GM’s prediction error dynamics.

### Tracking the free energy and deconflicting the generative model: the roles of affect and self-efficacy

In the AIF scheme, emotion and affect have been viewed as an organism’s experience of allostasis, and affective experience is understood as an interoceptive sensation of dyshomeostasis (homeostatic prediction error) in the context of relevant predictions (Seth et al., 2012; Barrett and Simmons, 2015). There is no consensus in the literature on the use of the terms affect, emotion, and feeling. Here, by affect, we mean a subjective valenced experience of allostasis, and by emotion – a contextualized affect. Frustration, for example, can be autonomic arousal with negative valence at the sight of missing a needed ride. In the present model, affect plays a role in deconflicting cognitive and physiological homeostatic GMs by providing domain-general sensory constraints on the precision of action policies through the system of self-efficacy (Figure 2). Affect is especially suited for this role because it integrates specialized channels of interoceptive signaling (such as heart and respiratory rates, blood pressure, electrolyte and glucose levels, etc.) into a binary choice of valence This arrangement makes for a nested hierarchy from the multitude of sensory streams to a generalized feeling of liking or disliking (affective valence) that informs and constrains the physiological homeostatic GM via the cognitive homeostatic GM as shown in Figure 2. It can be considered a recapitulation of the summation mechanism in bacterial taxis (Figure 1), where prediction error signals from multiple receptor clusters converge onto the effector protein determining the binary behavioral choice of the direction of the flagellar motor rotation. The integrating role of affect is necessary because having all the interoceptive sensory channels transparent to higher cognitive levels would overwhelm the GM, making the choice of behavior unreliable if not intractable. Even in bacteria, summation of sensory signals is necessary for choice behavior. But why need affect be consciously felt? Interoceptive information could be passed up the GM’s hierarchy unconsciously, which may be the case in simpler brains.^5^

Theories that ground affect and emotion in interoception explain the need for them to be conscious arguing that interoception, and by extension emotion, is inherent to consciousness and the sense of selfhood (Damasio, 1999; Seth et al., 2012). In the AIF theory, interoceptive inference generates emotion, “…emotional experience as arising from cognitive contextualization of changes in bodily states…” (Seth and Friston, 2016, 5). This does not mean that interoception is always conscious (most of it is not), but it does mean that once it is felt as an emotion, it becomes conscious. One can be (temporarily) unaware of his pain but cannot feel the pain without being conscious of it. The interoceptive view of emotion also addresses the conundrum of qualia (for review see Kanai and Tsuchiya, 2012). Just as “emotional experience arises from cognitive contextualization of changes in bodily states,” we suggest that change in cognitive states such as perception is contextualized by interoceptive experience, which confers a subjective quality, i.e., quale, on perception. Therefore, any conscious cognitive process that runs on a sentient platform will have qualia. It is challenging to obtain empirical support for this assertion,^6^ although consistent with it are findings that perceptual disturbances in derealization and depersonalization conditions are associated with decreased activity of the insula and anterior cingulate (Phillips et al., 2001), which indicates a possible disruption in the integration of physiological and cognitive homeostatic GMs (possible involvement of these brain regions in such integration is discussed in the next section).

Another property of affect making it useful for integrating physiologic and homeostatic GMs is that affect is a domain-general continuous variable that is felt in the present. This allows for integration over different timescales. Temporally deep counterfactual GMs, as the brain is, guide behavior on long-term scales, where the outcome is temporally removed from the decision on an action policy, and operate on predictions that can only be dis/confirmed in a distant future. Due to the temporal gap, regulating these models by immediate interoceptive signals would be an intractable task. Even the simple decision of chasing a bus (in the earlier discussed example) presents a challenge. The brain needs to estimate the expected surprise of being late relative to that of physiological dyshomeostasis of physical exertion (both having a high degree of uncertainty) in order to minimize its free energy. Yet, decision-making in humans often involves far greater timescales, such as enrolling in college, sometimes even surpassing a lifetime. Affect is always felt in the present and, therefore, can situate future counterfactual states in the context of present bodily and mental states. Selection and maintenance of long-term policies have to rely on the confidence of previously learned models (e.g., college = future success, college = fun, college = epistemic value, etc.). How is that confidence determined and regulated?

One possible mechanism is regulation by the system of self-efficacy (Figure 2). The reason we propose the mediating role of SE rather than direct regulation by affect is that affect is domain-general, which requires a mediator to target domain-, context-, and task-specific models and policies. In the absence of an immediate update of a GM through sensory input, the model is confined to the realm of cognitive homeostasis. At this level of abstraction, GMs run on epistemic input, and their policies are understood as sequences of mental actions (Metzinger, 2017). Cognitive homeostasis tracks a GM’s performance by the effect of selected mental acts on decreasing the model’s free energy. If the model performs well, it gets updated in a way that increases its confidence and vice versa. This raises the question of what a mechanism of such updating could be since, in the end, a given policy predicts a sensory outcome, not the free energy dynamics. We suggest that affective and SE dynamics could be that mechanism.

**Figure 3:**
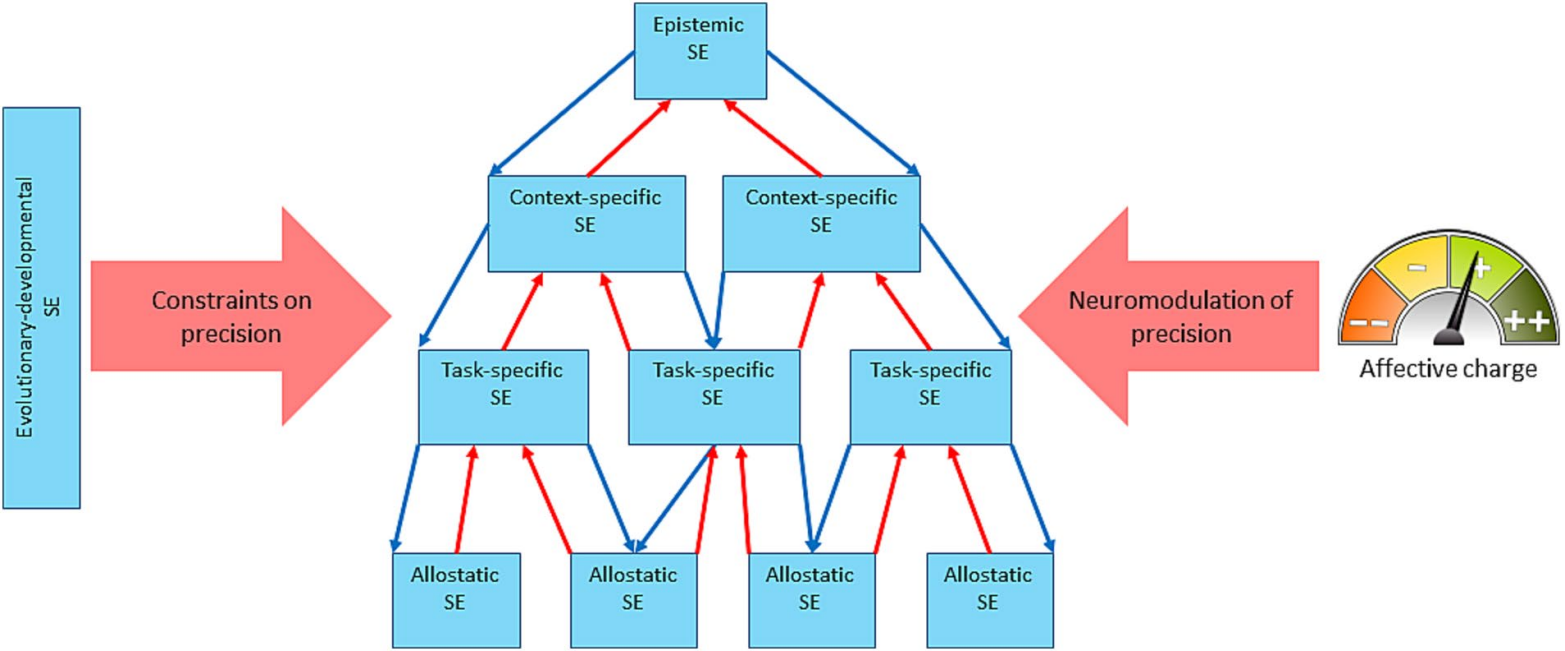
A schematic of the self-efficacy nested hierarchy and its regulation both intrinsically and by the affective charge. The self-efficacy (SE) hierarchy is nested under epistemic SE, descending to context-specific SE—task-specific SE—allostatic SE, with evolutionary-developmental SE exerting constraints on the precision of the whole hierarchy (the thick rose arrow). The affective charge modulates the precision of the SE system throughout the hierarchy (except the evolutionary-developmental SE which is phenotype- and developmental stage-dependent). The system autoregulates intrinsically via the counterflow of predictions on precisions (thin blue arrows) and prediction errors (thin red arrows).

From the standpoint of AIF (Joffily and Coricelli, 2013; Van De Cyrus, 2017; Kiverstein et al., 2019; Fernandez Velasco and Loev, 2021; Hesp et al., 2021) as well as reinforcement learning (Eldar et al., 2016), affective valence is the meta-cognitive mechanism of tracking cognitive performance. In this view, an accelerating rate of prediction error minimization feels good, whereas a decelerating one feels bad, which would also mean that dyshomeostasis feels bad and a return to homeostatic set points—good. This makes affect a subjective measure of the GM’s performance or, in the words of Hesp et al. (2021), “subjective fitness,” where subjective fitness is defined as the perceived efficacy of the organism’s GM. Affective dynamics track the GM’s performance on a moment-to-moment basis and average out as mood over larger timescales. As mental or actual acts change the GM’s level of surprise, its dynamics get sensory representation in affect dynamics which then regulate the model’s confidence/SE. This closes the integrated GM’s regulatory loop, making it an autoregulatory system (Figure 2) on any timescale. The putative mechanism of confidence regulation is by adjusting the model’s precision through neuromodulation of the synaptic gain (Feldman and Friston, 2010). SE can be conceived of as a nested hierarchy (Krupnik, 2020a, 2021, 2022) that comprises domain and task-general and specific levels, e.g., “I can do well in life”—“I can do well at physical fitness”—“I can run well”—“I can catch that bus.” It has to be noted that here we extend the original construct of SE as a person’s conscious subjective confidence that a chosen action will achieve the desired outcome (Bandura, 1977) to a more general concept. It includes model confidence at any level and considers it a probability function as in Friston et al. (2013). Such an extended concept of SE would, for example, include an organism’s unconscious confidence that increasing the heart rate will allow running faster. The SE hierarchy (Figure 3) can be conceived of as descending from epistemic SE (an agent’s confidence in its ability to know, or in AIF terms, a high precision of the self-model) to context-specific SE (confidence in inferences about a life-situation) to task-specific SE (confidence in actions in response to the situation) to allostatic SE (confidence in the body’s ability to execute the actions). The hierarchy is described as nested because it generalizes with every ascending step. For example, multiple physiological processes can support a single action; several actions may resolve a situation, etc. At the top, epistemic SE may apply to all domains of knowledge.

The notion of affect as a measure of subjective fitness can explain how purely mental acts may have affective valence. Solving a problem, figuring out a musical pattern, discerning the meaning of a text may result in decreased free energy and thus be indexed by a positive affect, the feeling of things “making sense.” Conversely, failure at such tasks may be associated with a negative affect. This also can explain emotional corollary to counterfactual mental images either from memory or fantasy since, for example, a memory of missing a bus would be contextualized by a currently felt affect.

The described here model can also offer an answer to the question asked at the beginning of this paper. Why do we often act in ways that make us feel bad or do not always act in ways that would make us feel good? The model has two components, one underwriting cognitive and the other—physiological homeostasis, where the cognitive homeostatic GM is hierarchically above the physiological one. They provide mutual constraints through the dynamics of affect. Being partially independent^7^ gives them a degree of autonomy in selecting action policies as long as they reduce the integrated GM’s free energy. Thus, in pursuit of decreasing *expected* surprise, the cognitive homeostatic GM may “force” an allostatic transition on its physiological subordinate. Such redundant regulation of allostasis can afford the agent greater flexibility in its behavior and, as a consequence, a more robust stress response. This is how exploratory behavior can happen, or how people’s desire to work out can be explained. For the goal of physical fitness (a state of expected low surprise), people can feel good about feeling tired and sore (a state of real-time physiological surprise). Then indeed, one can feel good about feeling bad. In the extreme case of suicide, a cognitive homeostatic GM can lead the organism to physical destruction (a physiological state of the highest surprise).

Outside extreme cases, the physiological homeostatic GM does not let the cognitive “run away.” In the earlier example with a bus, one would be unlikely to entertain the thought of being on time for a meeting in a state of starvation or dehydration. In case of conflicting “surprises,” policy selection is determined by the relative precision of the alternatives, such as catching the bus vs. relaxing and taking it easy. This view suggests a qualifying condition on Hohwy’s principle stating that in a hierarchical GM with competing hypotheses, the GM will choose one with the lower cumulative prediction error at the highest (most abstract) level of representation (Hohwy, 2013). In our model, such choice is constrained by the low-level sensory-near physiological homeostatic GM, and the “winning” hypothesis is one with the lowest cumulative prediction error for the whole (integrated) GM.

The stipulated role of affect in tracking a GM’s performance and thus regulating its confidence implies that policies associated with positive affect gain in confidence, and the person believes that he can because he likes. On the other hand, confident policies predict a decrease in the expected free energy with associated positive affect. The predicted positive affect can then be fulfilled through visceral AIF and, possibly. Later by the policy’s outcome. Therefore, one likes because he believes he can. Such an autoregulatory SE-affect loop implies that, unlike bacteria, in organisms complex enough to have it, behavior can be intrinsically rewarding as well as intrinsically aversive.

### Neural substrates of mutual control of cognitive and physiological homeostatic generative models

Neural pathways of mutual regulation between viscera and decision-making brain areas have been an area of extensive research. The key brain structures integrating interoceptive information with higher cognitive functions have been identified as the insula, anterior cingulate, and orbitofrontal cortices [as recently reviewed by Chen et al., 2021]. In the AIF theory of interoception (Gu et al., 2013; Seth and Friston, 2016; Paulus et al., 2019), the anterior insula issues predictions about the body’s internal states, which are compared to afferent visceral information, thus generating prediction errors. The errors are then resolved through autonomic reflexes as discussed above. The insula issues predictions based on exteroceptive information integrated and prioritized by the prefrontal and anterior cingulate cortices. The affective aspect of interoception emerges through signaling between the anterior insula and the affective-motivational network including the amygdala, ventral tegmental area, and ventral striatum. These brain circuits carry the bidirectional flow of information between exteroceptive and interoceptive GMs, which results in their mutual constraints and gives rise to emotions. In turn, affective valence is hypothesized to track the error dynamics of the integrated GM (Hesp et al., 2021). The implied function of such tracking is the regulation of the GM’s performance. It is, however, less clear what the neural mechanisms of such regulation are.

#### Closing the loop

The core hypothesis of the presented here model (Figure 2) is that dynamics of affective valence do not only reflect prediction error dynamics but regulate the confidence/precision of the model whose error dynamics they reflect. This also suggests that the learning function of affect is the regulation of the self-efficacy system. The idea that affective valence reflects the brain GM’s error dynamics for the purpose of emotional meta-learning has been developed by several researchers (Joffily and Coricelli, 2013; Van De Cyrus, 2017; Kiverstein et al., 2019; Hesp et al., 2021). In one version of this idea, change in the rate of prediction error minimization has been conceptualized as an “affective charge” which serves to update the precision of the very action model meant to minimize the prediction error (Hesp et al., 2021). Such a process has been formalized and tested in a computer simulation, but it remains unclear what neural mechanisms could embody it.

One of the challenges to mapping the process of updating the precision of a GM by its affective dynamics onto the brain is the hierarchical structure of the GM as well as the distributed circuits supporting it. The proposed here model envisions two parallel hierarchies, one of SE and the other of affect (Figure 3). The hierarchy of affect, as described in the referenced above literature, ascends through the sequence: interoceptive sensations—task-contextualized interoceptive sensations—context-contextualized (conscious) emotion—self-contextualized emotion—affective valence—affective charge. In our model, this contextualization first arises from integration of extero- and interoceptive GMs and at a higher level—from integration of cognitive and physiological homeostatic GMs (Figure 2).

The proposed here generalized notion of SE implies its widely-encompassing hierarchy, and its mechanistic meaning, as discussed above, is precision (model’s confidence) which is regulated through neuromodulation (Parr and Friston, 2017). An action policy comprises a hierarchy of mental and physical steps. It includes, at minimum, planning, interoceptive prediction, and physical and physiological action. Every component of this hierarchy has its precision. For a coherent and reliable behavior these precisions need coordination, which can be achieved via a nested hierarchy ascending through the sequence: allostatic—task-specific—context-specific—epistemic—evolutionary-developmental, where evolutionary-developmental properties of the brain provide constitutive constraints for the rest of the hierarchy (Figure 3). Using our reference example of chasing a bus, allostatic SE refers to the precision of an autonomic action policy at the sight of the bus ready to leave; the task-specific one refers to the precision of a motor action policy of running after it; the context-specific one refers to the precision of estimation of the expected utility of chasing the bus; the epistemic—to the precision of estimation of the utility/reliability of the estimate of the utility of the chase; and the evolutionary-developmental–to the precision of the organism’s internal affordances for all these acts (one needs to be endowed with legs and the skill of using them). It is easy to see how this action policy can disintegrate, should any of these precisions be out of proportion. For example, a low epistemic precision could result in indecisiveness despite the external and internal affordances to get on the bus; or an intrinsically low precision of motor control (such as in bradykinesia) may preclude the ride despite high precision of the other components of the hierarchy.

Our proposal that affect regulates the precision of action policies via the SE system is meant to explain how the dimensional domain- and task-general affective charge could regulate task- and context-specific policies. We suggest that the affective charge can bias the precision throughout the SE hierarchy while the relative precisions of its components are intrinsically regulated within the hierarchy, as hypothesized above (Figure 3). Phasic neuromodulatory activity of affective charge neurons coinciding in time with activity of action policy networks would ensure the specificity of the active charge’s effect. Tonic activity (corresponding to mood), on the other hand, would sustain the SE’s baseline state of precision. Thus, the dynamics of phasic vs. tonic activity of affective charge neurons could provide for fine-grained modulation of active inference by affect.

Given the wide span of the implied hierarchies, implementation of the hypothesized regulation of SE by affect would involve almost the whole brain. To add to the complexity, precision is regulated by neuromodulators, such as monoamines, that can have global as well as targeted effects (Likhtik and Johansen, 2019). One way to overcome the challenge of this complexity to elucidating neural mechanisms involved in the SE-affect loop could be to study mental phenomena and behaviors traceable to both affective inference and SE. Among the better-studied ones are fatigue and depression.

#### Fatigue

Fatigue, physical fatigue, in particular, is a suitable model behavior for studying the hypothesized regulation of self-efficacy by affect for several reasons. Within active inference, fatigue is viewed as a feeling state generated by the perceived dyshomeostasis that informs action policy selection and biases it toward rest and away from exertion (Stephan et al., 2016). Physical fatigue can be objectively measured by, e.g., maximal voluntary contraction (Taylor and Gandevia, 2008). Importantly, fatigue as a subjective experience of dyshomeostasis is distinguished from fatigability, its objective physiological manifestation (Kluger et al., 2013). Moreover, fatigue and fatigability can be experimentally dissociated by manipulating the physiology of peripheral muscles (making them fatigued) in the absence of actual work (Marcora, 2009). Such manipulation produces muscle dyshomeostasis without perception or a feeling of fatigue and presents an opportunity to differentiate the indirect effect (if any) of a subjective feeling of fatigue on the precision of relevant behavioral policies from the direct impact of interoceptive sensory input. The bottom-up manipulation of fatigability can be complemented by top-down manipulation of self-efficacy. For example, trained athletes compared to non-athletes showed higher resistance to fatigue as measured by physical effort discounting (Chong et al., 2018) and scored higher on measures of SE (Moritz et al., 2000). Interestingly, athletes were more tolerant of cognitive fatigue as well (Chong et al., 2018), implying a domain-general motivational mechanism, which is consistent with the idea of a wide range effect of affective charge on the SE hierarchy (Figure 3). SE can also be manipulated by false feedback with an ensuing effect on emotions (McAuley et al., 1999), suggesting an autoregulatory closed loop as in Figure 2.

The ability to dissociate fatigability from the subjective feeling of fatigue implies that fatigue is a function of inference on the expected exertion, and that fatigue, too, can be manipulated by false feedback. Independent manipulation of fatigue and SE helps address the question of causality, eloquently raised by Noakes,

“Vince Lombardi, the great American football coach, once wrote that: “Fatigue makes cowards of us all.” But he was wrong. For his arrow of causation points in the wrong direction. It is cowardice that exacerbates the sensations of fatigue, not the reverse” (Noakes, 2012).

This point of view is supported by the finding that manipulated SE can influence the feeling of fatigue (Jerome et al., 2002). There is also an observation that is consistent with the opposite direction of causation. A study of the role of the cerebellum in fatigue has shown that fatigue (defined by the authors as “perception of fatigue”) may affect motor control by the cerebellum (Agostina et al., 2023). This finding can be interpreted as an indication that fatigue influences the precision/SE of motor policies in the cerebellum. Our AIF-based model suggests that the causation arrow is a circle (Figure 2); the feeling of fatigue exacerbates cowardice (low SE) which infers greater expected exertion entailing greater fatigue.

Physical fatigue as a model behavior has the advantage of a clear endpoint in the GM’s hierarchy, which is premotor and motor cortices. Multiple lines of evidence indicate that the subjective feeling of fatigue is not generated by afferent sensory input from the viscera and skeletal muscles but by central inferential processes (Marcora, 2009). This is consistent with the aforementioned AIF theory of fatigue (Stephan et al., 2016) and indicates that fatigue can affect decision-making by inflating the value of the expected effort. This hypothesis was directly tested in a neuroimaging study of choice behavior under conditions of physical exertion (Hogan et al., 2020). The authors demonstrated that subjective fatigue affected the evaluation of the prospective effort as well as the decision to apply it by inflating the effort value and decreasing the commitment to it. They also identified brain structures involved in this decision-making and showed that fatigue inhibited premotor cortex’ activity. A decrease in this activity was, in turn, signaled to the insula, thus increasing the prospective effort value. Effort value was found encoded in the anterior cingulate and ventromedial prefrontal cortices (Aridan et al., 2019; Hogan et al., 2019, 2020). Together, these data are consistent with the emerging view of the allostatic GM regulating exertion (physical and mental) and decisions about putting forth effort. This regulation is instantiated by the network of prefrontal, anterior cingulate, insular, and premotor and motor (for physical effort) cortices (Müller and Apps, 2019; Paulus and Khalsa, 2021; Greenhouse-Tucknott et al., 2022). In this view, the insula serves as a point of integration of affective, evaluative, and interoceptive information it receives from the salience network, prefrontal cortex, and visceral afferents, respectively. It also places the “cowardice-fatigue” circle of causality within the insula.

The described scheme is a simplification considering the rich and diverse contexts where real behavioral choices happen. For example, behaviors can be planned on different timescales, they are conditional not only on effort value but environmental affordances on different levels (from energy resources to social); behaviors can be automatic, deliberate, or novel. Indeed, a recent study implicated the cerebellum in the regulation of physical fatigue and demonstrated that fatigue affects cerebellar excitability and cerebellum-dependent task performance (Agostina et al., 2023). From the reviewed work, it follows that subjective feeling fatigue can influence SE at different levels of its hierarchy, which is consistent with our model (Figure 3). The model also suggests that the impact of affect on SE depends on the temporal proximity of an action policy and change in affect. This offers a testable prediction that “scoring” in a physical (e.g., sports) or mental (e.g., problem solving) endeavor is an effective way to not only boost SE but alleviate the fatigue.

There are systemic clinical conditions related to pathological fatigue including chronic fatigue syndrome and depression. Interestingly, one of the common features of depressive disorders is inflated effort value (Vinckier et al., 2022). It can explain the pervasive deficit of motivation in depression, and fatigue being one of its symptoms (*Diagnostic and Statistical Manual of Mental Disorder,* 5th ed. text rev; *DSM-5*; American Psychiatric Association, 2022).

#### Depression

Depression has been conceptualized as a disorder of inference by several researchers (reviewed recently in Gilbert et al., 2022). Some of these theories focused in particular on dysregulation of allostasis as the basis of depressive etiology (Barrett et al., 2016; Stephan et al., 2016; Arnaldo et al., 2022). As a disorder of allostasis, the depressed mind is seen as “locked-in” the process of chronically predicting and fulfilling dyshomeostasis at all levels of behavioral hierarchy from humoral regulation of metabolism and physiology to abstract decisions about engaging with the outside world (Barrett et al., 2016). Along the same line, the active inference theory of fatigue considers depression as generalized fatigue stemming from allostatic self-inefficacy (Stephan et al., 2016). More specifically, a depressed agent believes in its failure to decrease interoceptive surprise. A belief in the incompetence of one’s GM then leads to behavioral withdrawal as in learned helplessness and to a sense of hopelessness. Interestingly, when bacteria find themselves in a state of chronic dyshomeostasis due to poor environmental conditions, they undergo sporulation which can be seen as metabolic withdrawal as it puts them in a state of dormancy (De Hoon et al., 2010).

Building on the idea of self-efficacy as a metacognitive regulator of allostasis and its role in depression, SE was proposed as a metacognitive regulator of the depressive type of stress response (Krupnik, 2020a, 2021). We expand this idea here within the proposed model of the role SE plays in cognitive and physiological homeostasis. A common theme emerging in discourse about the depressive GM is its failure of confidence and its inability to make accurate inferences about the world. This idea has been aptly expressed by Clark et al. (2018, p. 2,278), “Major depression occurs when the brain is certain that it will encounter an uncertain environment, i.e., the world that is inherently volatile, capricious, unpredictable and uncontrollable.”

Our model, specifically the partition into cognitive and physiological homeostatic GMs (Figure 2), helps explain the stability of depressive GM and its resistance to change and therapy. At first glance, the depressive GM may appear prone to instability and decay as it is failing at self-evidencing due to its agnostic nature: perceiving the world as unpredictable and uncertain, which could lead to high surprise and, consequently, high free energy. The above quotation, however, implies that the depressive GM can depress its free energy by way of a highly precise belief in its certainty about the world’s uncertainty which then becomes expected and unsurprising. This certainty may serve as a metacognitive clamp on the GM’s free energy. In addition, at the physiological level, the GM predicts its inefficacy in resolving dyshomeostasis (Stephan et al., 2016). Provided cognitive and physiological homeostatic GMs’ mutual constraints, it can be seen how they can reinforce each other’s depressive inferences. A belief in one’s inability to control the external environment would predict inescapable stress down to the physiological homeostatic GM which, in turn, will fulfill that prediction through autonomic AIF, sending upward confirmatory sensory information of physiological stress in the form of a negative affective charge. This way, the depressive GM can recurrently self-evidence, gaining in stability. We propose rumination as the cognitive mechanism of such self-evidencing.

Rumination (negative/depressive) is a hallmark of depression (Nolen-Hoeksema et al., 2008), manifesting in self-referential circular thinking about the causes of negative experiences and events. Multiple models have been suggested to explain rumination (Smith and Alloy, 2009), and the debate about its mechanisms and function is ongoing. Depressed people feel compelled to engage in rumination despite its association with negative affect (Nolen-Hoeksema et al., 2008). To resolve this paradox, the idea of rumination’s functional utility as a problem-solving process has been set forth. From the evolutionary perspective, the “analytical rumination” hypothesis was developed, where rumination was regarded as the organism’s attempt at finding a solution to the predicament that caused its depression (Andrews and Thompson, 2009). Analytical rumination is hypothesized as a mechanism for concentrating resources on the trigger problem and disengaging from all other mental and physical engagements. In AIF, rumination has been framed as repetitive sampling of behavioral policies meant to help the organism out of its depressive predicament (Berg et al., 2022). The reason for compulsive and repetitive sampling is believed to lie in its ineffectiveness due to the sampling bias. In this view, the depressed state biases the organism toward resampling the same failing policies in a perpetual cycle.

We propose an alternative AIF hypothesis, where the function of rumination is not actual or attempted problem-solving but self-evidencing by the depressive GM. Rumination is a mental action that repeatedly confirms the GM’s inference about the world as defeating and about itself as defeated (low SE). Such inference is confirmed by the physiological homeostatic GM predicting inescapable stress and confirming it through autonomic AIF. Thus, rumination perpetually suppresses/explains away the prediction error generated by chronic dyshomeostasis. As a result, the depressive GM is trapped in the cycle of reducing its free energy by rumination, while the energy is raised by dyshomeostasis.

This analysis is incomplete without considering the role of affect in the ruminative cycle. According to our model, the negative affective charge associated with rumination is expected to decrease the depressive GM’s confidence and thus destabilize it, which contradicts our explanation of the model’s stability. The contradiction can be resolved by taking into account that at the highest, epistemic level, rumination increases the (self) model’s confidence by “explaining” its inefficacy (“I know that (and why) I am inefficacious”). However, according to the AIF theory of affect, an increase in a model’s confidence is indexed by positive not negative affect. Our hypothesis predicts *both* negative and positive affective reactions within the ruminative cycle. Like any completed mental action, it starts with an increase in surprise (indexed by negative affect) and resolves in its decrease (indexed by positive affect). Still, many studies (reviewed in Watkins, 2008) have shown that rumination does not just “tread water” of depressed mood but contributes to its onset and deepening. In our view, as rumination continues the self-evidencing of the depressive GM, it engages a broader scope of negative cognition and promotes a deeper behavioral withdrawal, thus focusing away from opportunities (cognitive and behavioral) to generate positive affect. Therefore, the net affective balance of prolonged rumination is expected to be negative.

Rather than a state, depression is a dynamic process with its intrinsic logic and dynamics, as evolutionary theories of depression have argued since long ago (Kaufman and Rosenblum, 1967) and as has also been emphasized from the predictive processing perspective (Krupnik, 2020a, 2021). The importance of considering the natural dynamics of the depressive stress response has been reflected in an evolutionary- and acceptance-based therapy for depression (Krupnik, 2014). It suggests that interventions need to be precisely timed and specific to the stage of the depressive process. More specifically, the initial phase of depression is seen as a progression toward acceptance of the organism’s defeat through emotional and behavioral withdrawal (Watt and Panksepp, 2009). We suggest that rumination is instrumental in this process as it works toward confidence in the depressive GM’s inefficacy, which is the AIF meaning of learned helplessness (Stephan et al., 2016). Previously, we suggested that this phase was a cognitive dead end that was supposed to bottom out, which in therapeutic settings, could be aided with acceptance-based interventions (Krupnik, 2021). The proposed here SE model can explain why this may be the case. As the depressive GM keeps gaining in epistemic confidence about its inefficacy, the inefficacy itself decreases in its precision weight as a prediction error, thus becoming unsurprising, and stops sequestering attention and mental resources thus relaxing the constraints of the ruminative cycle. At this point, the depressive GM is expected to become more amenable to generating positive affect and to other interventions such as standard cognitive behavioral therapy and behavioral activation.

The present model suggests that the affect-SE loop is an important target for both research and therapy. SE and affect can be manipulated; SE, as mentioned earlier,—by false feedback, and affect—by exposure to salient emotional stimuli as well as chemically with opioids (Nummenmaa and Tuominen, 2018). Manipulation of the opioid system may be especially interesting to study since activation of the μ-opioid receptor leads to a release of dopamine by dopaminergic neurons of the ventral tegmental area (McGovern et al., 2023), and dopamine can affect precision and, therefore, the model’s confidence by modulating synaptic gain (Friston et al., 2012). Moreover, endogenous opioids can modulate synaptic gain directly (Winters et al., 2017) representing a parallel dopamine-independent mechanism of regulating precision. This is consistent with our model, where affect regulates the GM’s precision via SE (Figure 2). The wide distribution of opioid receptors and opioidergic neurons in the brain (Le Merrer et al., 2009; Reeves et al., 2022) is consistent with our hypothesis about a widely distributed effect of the affective charge on the SE hierarchy (Figure 3).

The opioid system has been implicated in depression in both animal models and humans (Peciña et al., 2019). Opioids have shown an acute anti-depressant effect (Gerner et al., 1980; Nyhuis et al., 2008) similar to that of ketamine. Moreover, ketamine potentiates endogenous opiate signaling (Gupta et al., 2011), which could partially explain its antidepressant activity in addition to (or as part of) its NMDA-mediated effect since an antagonist of μ-opioid receptor attenuates ketamine’s antidepressant effect (Williams et al., 2018). Interestingly, genetic studies of μ-receptor variants in humans linked its possible role in depression to opioidergic activity in the anterior cingulate and anterior insula (Way et al., 2009), the areas responsible for allostatic regulation and the hypothesized cross-talk between physiological and cognitive homeostasis, as discussed earlier. Continuation of these lines of research may establish a detailed map of the effects of affect dysregulation on the depressive GM, as well as advance therapeutics for depressive disorders.

In summary, the present model emphasizes the role of mutual regulation by the physiological and cognitive homeostatic GMs in the maintenance and dynamics of the depressive GM. Rumination and affect-dependent SE emerge as core cognitive mechanisms of these dynamics, where the affect-SE autoregulatory loop (or the “like-can-like” loop) can, under certain conditions, devolve into depression. This view brings SE into focus for both research and therapy. Depression is a systemic condition, which presumes a systemic approach to its therapy. Indeed, combining cognitive, behavioral, and chemical interventions has become the mainstay in treatment of depression. However, SE has not been specifically and explicitly targeted in therapy of depression or in psychotherapy in general. It has recently been identified as a possible target for development of the therapeutic alliance (Krupnik, 2022), but the above formulation of depression suggests that interventions targeting SE could be a valuable addition to the anti-depressant toolkit.

## Conclusion

Here, we present an active inference framework for bacterial chemotaxis which serves as a mechanism of homeostasis of the simplest living system. A core feature of this mechanism is the mutual constraint between extero- and interoception in the regulation of bacterial motion. We extrapolate this principle to more advanced organisms including humans by partitioning the mechanism of homeostasis into cognitive and physiological homeostatic GMs. The resulting AIF model of homeo- and allostasis represents a closed autoregulatory loop between the two GMs, where the physiological homeostatic GM is based on integrated extero- and interoceptive GMs, and the cognitive homeostatic GM—on the interaction of hierarchical affective and self-efficacy systems. Prediction error dynamics and precision control are proposed as the mechanisms of integration of the physiological and cognitive homeostatic GMs into the integrated homeostatic GM (Figure 2).

Two related cognitive-physiological phenomena, fatigue and depression, are identified as promising targets for studying the neural mechanisms of the proposed model. Both are involved in emotional and homeostatic regulation and rely on partially overlapping brain circuitries. A central question for such studies is the structural organization, neurochemistry, and dynamics of the homeostatic GM’s regulation by the affective charge. A related question is how prediction error dynamics are translated into the neurochemistry of affect and its valence.

In their article on allostatic SE, the authors ponder, “This raises the interesting question what, ultimately, the highest set point or belief is that dictates the behavior of individual humans” (Stephan et al., 2016, p. 22). The answer implied by our model is that the highest set point is epistemic SE (Figure 3), confidence in the validity and accuracy of one’s cognition, or simply put, “I know that I can know.” The epistemic SE model perpetually self-evidences by decreasing the brain’s free energy, thus integrating its component models into one coherent GM. It is ever at work since the free energy cannot be kept steady due to the ever-dynamic internal and external worlds.

“I like therefore I can,” in pathological fatigue and depression, devolves into a spiraling vicious cycle “I do not like it therefore I cannot—therefore I will not like it—therefore I will fail at it—therefore I will not like it…” Simpler organisms such as bacteria do not possess the affect-SE loop and cannot suffer from depression but, on the flip side, they cannot have intrinsically rewarding behaviors either.

## Data availability statement

The original contributions presented in the study are included in the article/supplementary material, further inquiries can be directed to the corresponding author.

## Author contributions

VK: Conceptualization, Writing – original draft.

## Figures and Tables

**Table 1:** 

Homeostasis	A process by which a system maintains its parameters close to their optimal value (set points).
Allostasis	A process by which a system anticipates and changes the value of its parameters to meet the demands of the environment. Allostasis results in deviation of the system’s parameters from its set points.
Allostatic load	A measure of a system’s deviation from its homeostatic set points. It also corresponds to the level of stress.
Affect	An organism’s subjective experience of allostasis.
Nested hierarchy	A hierarchical system where several elements at one level are subordinate (nested under) to fewer elements at the next level up. A complete nested hierarchy is a pyramid with multiple elements at its base nested under one element at the top.
Integration	A process whereby two (or more) systems become partially interdependent through mutual constraints and regulation, thus forming a new integrated system.
Generative model (GM)	A model that generates predictions (in the form of probability distribution) about sensory input it receives from the environment. These predictions are based on the model’s prior beliefs (priors) about the environment’s causal structure, that is, what events of event sequences cause what sensations. The model updates itself (its priors) by minimizing the mismatch (prediction error) between predicted and actual sensory input.
Self-evidencing	By minimizing prediction errors, a GM confirms itself or maximizes evidence of itself.
Exteroceptive GM	A GM that generates predictions about the organism’s external environment.
Interoceptive GM	A GM that generates predictions about the organism’s internal (excluding the brain) environment.
Active inference (AIF)	A theory, part of the predictive processing paradigm, that explains behavior as minimization of prediction errors and variational free energy.
Variational free energy	An information theoretic quantity that is the upper bound on the GM’s surprise associated with prediction errors.
Affective charge	An organism’s subjective experience of prediction error dynamics.
Physiological homeostasis	A process by which an organism maintains its physiological parameters close to their optimal value (set points).
Cognitive homeostasis	A process by which a brain minimizes its variational free energy.
Homeostatic GM	A GM that has the system’s set points as its priors, and predicts the system’s parameter values to be close to the set points.
Allostatic GM	A GM that predicts change of the system’s parameter values in accordance to the environmental demands.
Physiological homeostatic GM	A brain GM that generates predictions about the organism’s physiological parameter values such as heart rate, blood pressure, glucose levels, etc.
Cognitive homeostatic GM	A brain GM that generates predictions of decreasing variational free energy.
Self-efficacy (SE)	A GM’s confidence in its predictions or inferences. Quantitatively, SE corresponds to the GM’s precision.
SE hierarchy	A hierarchical organization of confidences (or precisions) at different levels of the GM. It descends from epistemic SE (confidence in the GM’s validity or accuracy) to contextual SE (confidence in the GM’s predictions about a situation) to action SE (confidence in the GM’s action policies in the situation) to allostatic SE (confidence in the GM’s within-the-body-action policies supporting its overt action policies).

## Footnotes

^1^We use the terms stress and dyshomeostasis interchangeably.

^2^Separated by Markov blankets.

^3^Interoceptive GM can be viewed as comprising a proprioceptive GM controlling motor movement and a visceral GM controlling allostasis. To keep this paper focused, we leave the discussion of the proprioceptive GM out.

^4^The concept of cognitive homeostasis does not imply that physiological one is not cognitive but points at the different nature of their variables and set points. It could be argued that physiological homeostasis is not cognitive but computational, but there is no consensus regarding the conceptual brackets between computational and cognitive.

^5^There is no consensus about where in the phylogenetic tree conscious feelings first emerged.

^6^It may be possible to get a more definitive answer in the future when/if artificial intelligence develops to the level of being sentient *and* able to communicate its subjective experience.

^7^Separated by Markov blankets.
